# Translation and psychometric evaluation of the persian version of the “Nursing Profession Self-Efficacy Scale”

**DOI:** 10.1186/s12912-023-01182-3

**Published:** 2023-01-25

**Authors:** Zahra Lazemi, Maasoumeh Barkhordari-Sharifabad

**Affiliations:** Department of Nursing, School of Medical Sciences, Yazd Branch, Islamic Azad University, Shohadaye Gomnam Blvd., Safaiyeh, 8916871967 Yazd, Iran

**Keywords:** Validity, Reliability, Self-efficacy, Nurses, Nursing profession, Questionnaire

## Abstract

**Background:**

Self-efficacy demonstrates the general competence of nurses in providing nursing care. Evaluation of nurses' self-efficacy is of particular importance to improve nursing care. The existing self-efficacy scales in Iran are insufficient due to lack of focus on the specific issues of the nursing profession. This study was conducted with the aim of translating and psychometrically evaluating the Persian version of "Nursing Profession Self-Efficacy Scale" (NPSES).

**Materials and method:**

This cross-sectional study was conducted in two parts: translation and psychometric evaluation. After getting permission from the original developer of the scale, the process of translating the questionnaire into Persian was done based on the Polit and Yang model. Then, the face validity was explored using a qualitative method with the participation of 10 nurses. The content validity was investigated using a qualitative and quantitative method (content validity index) with the participation of 15 experts. Construct validity was surveyed with exploratory and confirmatory factor analysis via completing the questionnaire by 550 nurses worked in different wards of hospitals affiliated to Shahid Sadoughi University of Medical Sciences, Yazd/Iran, who were selected using convenience sampling. The reliability of the tool was also established with internal consistency and stability methods, with the participation of 30 nurses. SPSS20 and AMOS were used for data analysis.

**Results:**

"Nursing Profession Self-Efficacy Scale" includes 19 items for which the translation and cultural compatibility of the Persian version were confirmed. Face validity and content validity (SCVI/Ave = 0.9) were acceptable. By conducting exploratory factor analysis, three factors (professional situation, care situation, and support situation) were identified, which accounted for 62.38% of the variance of the scores. In the confirmatory factor analysis, the values of the fit indices confirmed the appropriate fit of the model. The reliability was established using Cronbach’s α method (α = 0.86) and an ICC of 0.83, indicating that the scale was reliable.

**Conclusion:**

The translation, validity, and reliability of the Persian version of "Nursing Profession Self-Efficacy Scale" suggested that this tool has a clear and legitimate translation. Also, this tool has good validity and reliability and can be used as one of the tools to measure the self-efficacy of Iranian nurses.

## Background

Nurses, as a large part of healthcare staff, cover a major part of health-related services and with their behavior and performance, play an essential role in providing quality care [1]. Among the key missions of nurses is to understand the needs and interests of community and to promote a safe and effective environment in the administration and promotion of health policies. Recorded information indicates that if nurses do not play an active role as members or coordinators of the treatment team, provision of medical care will face some difficulties [2]. Nurses are required to provide quality and satisfactory services for patients. To perform each task, they must have certain behavioral tendencies [1]. One of these behavioral tendencies is self-efficacy, which affects the performance of employees [3].

Self-efficacy theory acquires its roots from Bandura's socio-cognitive theory [4], which emphasizes the beliefs that people have about their abilities to solve problems and challenges in certain situations [5]. In this theory, self-efficacy is one of the personal beliefs that people need for success and it can be an indicator of a person's manner of thinking and acting [6]. Bandura defines self-efficacy as people's belief in their ability to perform desired functions, and considers it the determining factor of how people think, behave, and feel [7]. Bandura has acknowledged that four important factors play a role in the emergence and promotion of self-efficacy. These factors include: mastery experiences or performance outcomes, vicarious experiences or social patterns, social encouragement or verbal persuasion, and emotional or physiological states [7].

Self-efficacy is a known concept that affects nurses' beliefs, actions, and behaviors while caring for patients [8]. It plays an important role in nurses' motivation for care, decision-making, prioritizing interventions, and encouraging them to continue caring for patients despite problems and failures [9]. Increased self-efficacy can lead to high-quality nursing care and improve individual and organizational performance [10]. Bandura's self-efficacy theory has been repeatedly researched in nursing clinical settings and the results of existing studies confirm the appropriate application of this theory in clinical settings [11]. Nursing research has revealed that nurses who believe in high self-efficacy consider obstacles as an opportunity instead of a threat [12]. Nurses’ self-efficacy affects the quality of their work. Nurses with low self-efficacy often do not have enough self-confidence and this affects the quality of care [13]. In their study, Zulkosky et al. stated that nurses who believe in their clinical ability and effectiveness and consider it efficacious and useful will have better mood and mental ability [14]. Moreover, Manojlovich's study showed that self-efficacy plays a mediating role between the structural strength and professional performance of nurses and recommends nursing managers to improve nurses' professional performance behaviors by creating opportunities to strengthen nurses' self-efficacy [15]. Kurnia et al.'s study showed that nurses should have high self-efficacy to provide quality palliative care for patients and their families [16].

To improve nurses' self-efficacy, their self-efficacy level should be evaluated first [17]. Yet, it should be noted that the self-efficacy of an individual to perform work in a professional field may be very different depending on the desired profession [18, 19]. The use of general questionnaires to evaluate the self-efficacy of the nursing profession can affect the accuracy and correctness of the results and weaken it [20]. This is consistent with Bandura's theory, which suggests that self-efficacy beliefs are behavior- and situation-specific. Bandura states the basic principle that "the content of self-efficacy scale items should express beliefs about individual abilities to determine specific levels of performance” [7]. Since the range of skills required for nursing is different [21], therefore, to evaluate professional self-efficacy, special evaluation tools should be developed for the profession [20].

The existing self-efficacy scales in Iran are insufficient due to lack of focus on the specific issues of the nursing profession. Many studies that evaluate the self-efficacy of health care professionals, including nurses in Iran, have been conducted using Sherer's general self-efficacy scale [22]. Besides, the Clinical Performance Self-efficacy Questionnaire was developed in Iran by Cheraghi et al., which measures the self-efficacy of nursing students [11]. One of the existing tools to evaluate self-efficacy of nursing profession is the Nursing Profession Self-Efficacy Scale (NPSES), which was developed by Caruso et al. in Italy [18]. This scale is based on Bandura's theory. It assesses nurses’ general confidence in coping with daily challenges. This scale has two dimensions of characteristics of nursing situations and professional situations [18]. This scale was investigated in South Korea by Oh et al. for validity and reliability; the results showed that it is a suitable psychometric instrument for use in the clinical environment of Korea [20].

Iran is a developing country and the nursing workforce in Iran is estimated to be 150,000 at different levels. The healthcare system in Iran, like other countries, faces challenges such as lack of human resources and job dissatisfaction [23, 24]. Considering the similar environmental conditions of nursing in Italy and Iran, such as difficult working conditions [18], shortage of nurses, and high ratio of nurses to patients [24–27], NPSES can be a potentially valid tool to be used for Iranian nurses. Since this tool is used to check nurses' confidence in dealing with job challenges, its validation in the Iranian context can have a major impact on nursing management. Also, using a valid specialized index to evaluate the level of self-efficacy of Iranian nurses can lead to more accurate and efficient results. Thus, this study was conducted with the aim of psychometrically validating the Persian version of the special self-efficacy tool of nurses developed by Caruso.

## Methods

### Study design

This cross-sectional study included two stages. In the first stage, the translation and cultural adaptation of the tool was done; in the second stage, the psychometric evaluation of the instrument was performed.

- Stage I: Translation and cultural adaptation
  In so doing, after obtaining permission from the original developers of the "Nursing Profession Self-Efficacy Scale", the process of translation and cultural adaptation was carried out based on the model of Polit and Yang [28].
  - *Forward translation:* Based on this model, the translation of the tool from English into Persian was done independently by two Iranian translators who were fluent in Persian and English languages and culture.
  - *Combination of early translations (synthesis):* Persian translations were reviewed in the presence of experts to create a single translation.
  - *Back-translation:* In the next stage, the Persian translation was back-translated into English again by two other translators, fluent in both Persian and English languages, without knowing the main items of the tool.
  - *Reconciliation:* With the consultation and opinion of experts, the distilled version that was back-translated into English was agreed upon.
  - *Pre-testing and cognitive interviewing:* In order to test the tentative final version, 10 nurses were asked to provide us with their opinions on the difficulty, irrelevance and ambiguity of each item (qualitative face validity).
  - *Final version:* Finally, the final revised version was sent to the main developer of the tool for feedback, which was approved by him.
- Stage II: Psychometric testing
  1. Validity
    *Face validity:* The instrument translated into Persian was given to 10 nurses to determine the face validity using a qualitative method, and the items were examined in terms of difficulty level, diction and wording ambiguity, and appropriateness level [29].
    *Content validity:* In the next step, to evaluate the validity of the content, using a qualitative and quantitative method (content validity index), 15 professional nursing professors and experts in the field of psychometrics were asked to give their opinions about the relevance of items to the intended concept and use of appropriate diction and wording. After careful study of their comments, appropriate corrections were made by the research team. If the score of the content validity index of the scale was higher than 0.79, then the content validity of the scale was confirmed [30].
    *Construct validity (exploratory and confirmatory factor analysis):* In the present study, construct validity was investigated using exploratory and confirmatory factor analysis.
      To determine construct validity (factorial analysis), 3 to 10 people are needed for each item in the instrument [31]. In this research, 350 nurses participated in exploratory factor analysis and 200 nurses participated in confirmatory factor analysis. Participants worked in different wards of hospitals affiliated to Shahid Sadoughi University of Medical Sciences, Yazd/Iran. Nurses who met the inclusion criteria were selected using convenience sampling. The inclusion criteria were: holding at least a bachelor's degree in nursing, at least six months of work experience in treatment wards, and willingness to participate in the study.
      To confirm the adequacy of the sample, the Kaiser–Meyer–Olkin (KMO) test and Bartlett's sphericity test were used to extract the factors. KMO index was equal to 0.921. A KMO value higher than 0.5 is acceptable [32, 33]. Bartlett's test was significant (*P* < 0.001). These results indicated that the data set was suitable for factor analysis.
      EFA was performed by principal component analysis followed by varimax rotation. Eigen values and factor loadings were considered higher than 1 and 0.4, respectively [34].
      Then, the confirmatory factor analysis was used to confirm the dimensions of the questionnaire and the proposed model of exploratory factor analysis. In this study, indices of fit of *χ*^*2*^/degree of freedom (df), Root Mean Square Error of Approximation (RMSEA), Comparative Fit Index (CFI), Normed Fit Index (NFI) [35].
  2. Reliability
    The reliability was examined by the method of internal consistency and stability (Cronbach's α coefficient). To establish the reliability of stability, 30 participants completed the Persian scale with an interval of 2 weeks [36], and then the scores obtained were compared with the intra-class correlation test. To interpret the results, Cronbach's α and ICC values higher than 0.7 are considered satisfactory [37].

### Data collection and analysis

The tools used in data collection were demographic information questionnaire and Nursing Profession Self-efficacy Scale.

Demographic information questionnaire was used to obtain information in personal and professional fields including: gender, marital status, level of education, ward of service, total employment history, and employment history in the current ward.

"Nursing Profession Self-Efficacy Scale" included 19 items wherein each item is scored on a five-point Likert scale (from ‘not at all confident’ to ‘completely confident’); a higher score indicates higher self-efficacy. This scale includes two dimensions of attributes of caring situations (12 items) and professionalism situations (7 items). Face, content, and construct validity as well as concurrent validity of the original scale have been examined. Cronbach's alpha was 0.83 for overall scale [18].

Data were collected during December 2021 to May 2022. The coded data were analyzed by SPSS20 and AMOS.

## Findings

### Stage I: Translation and cultural adaptation.

At the beginning of the study, the scale was translated in a standard way in several steps. The findings indicated the acceptability of the translation of the original scale into Persian.

### Stage II: Psychometric testing

#### Validity

##### Face validity

After examining the opinions of the nurses, due to nurses’ difficulty with understanding of the concept, changes were made in items 5, 7, and 19, for a better understanding of the concept, and the questionnaire was given to the same nurses again.

##### Content validity

The content validity index for all items ranged from 0.8–1.0. Accordingly, none of the items were removed. The average content validity index (S-CVI/Ave) was obtained as 0.9.

##### Construct validity

**- Sample characteristics**

Three hundred fifty nurses participated in exploratory factor analysis and 200 nurses participated in confirmatory factor analysis. Based on the demographic characteristics of the participants in the study, the average age of the nurses studied was 33.68 years with an age range of 23 to 50 years. Among the subjects studied, 332 (60.4%) were female (allocation of code “1” for female and code “2” for male), 355 (64.5%) were married (singles were assigned a code of “1” and married were assigned a code of 2), 499 (90.7%) had a bachelor's degree (allocation of codes 1–3 for BS, MSc, and PhD, respectively) (Table 1). No significant statistical difference was found between the demographic characteristics of the participants in the exploratory and confirmatory factor analysis.

**Table 1:** Demographic characteristics of the participants

Variables	Levels	Total sample (*N* = 550)	Exploratory (*N* = 350)	Confirmatory (*N *= 200)
		***N*** **(%)**	***N*** **(%)**	***N*** **(%)**
Gender	Female	332 (60.40)	211 (60.30)	121 (60.50)
	Male	218 (39.60)	139 (39.70)	79 (39.50)
Marital status	Single	195 (35.50)	105 (30.00)	90 (45.00)
	Married	355 (64.50)	245 (70.00)	110 (55.00)
Education level	BS	499 (90.70)	322 (92.00)	177 (88.50)
	MSc	47 (8.50)	26 (7.42)	21 (10.50)
	PhD	4 (0.80)	2 (0.58)	2 (1.00)
Working ward	CCU, ICU, NICU, Pediatric ICU	188 (34.18)	112 (32.00)	76 (38.00)
	ER	78 (14.18)	42 (12.00)	36 (18.00)
	Dialysis	17 (3.09)	10 (2.86)	7 (3.50)
	Burns	24 (4.36)	16 (4.57)	8 (4.00)
	Internal	103 (18.73)	78 (22.28)	25 (12.50)
	Surgery	42 (7.64)	26 (7.42)	16 (8.00)
	Orthopedics	28 (5.09)	21 (6.00)	7 (3.50)
	Infectious diseases	22 (4.00)	14 (4.00)	8 (4.00)
	ENT	18 (3.27)	8 (2.26)	10 (5.00)
	Pediatrics	30 (5.45)	23 (6.57)	7 (3.50)
**Variables**		**mean (SD)**	**mean (SD)**	**mean (SD)**
Age (year)		33.68 (6.84)	34.76 (6.91)	31.79 (6.31)
Work experience (year)		10.56 (7.31)	11.28 (6.71)	9.29 (8.13)
Work experience in current ward (month)		49 (49.02)	55.64 (55.30)	39.06 (33.02)

**- Exploratory factor analysis**

**Fig. 1:**
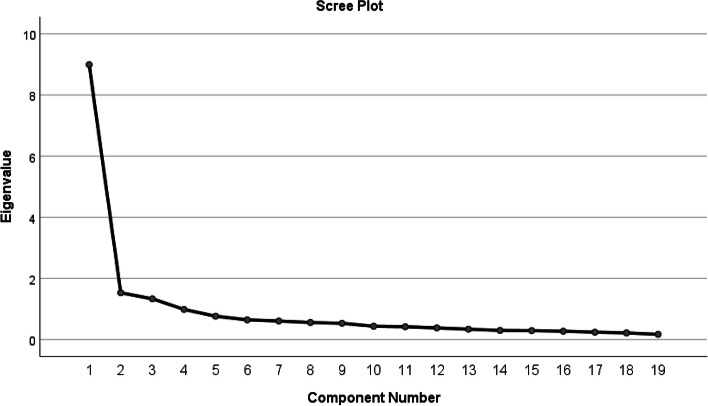
Scree plot for the Persian version of the "nursing profession self-efficacy scale"

Principal component analysis was used to extract factors, and Eigenvalue method and scree plot were used to determine the number of factors. Based on the Eigenvalue above 1 and the scree plot, three factors (professional situation, care situation, and support situation) were extracted for the nursing profession self-efficacy scale, which accounted for 62.38% of the total variance (Table 2, Fig. 1).

**Table 2:** Extracted factors, variance, and number of items of each factor

Factors	Percentage of explained variance	Percentage of cumulative explained variance	Number of items
Factor 1: Professional situation	47.32	47.32	8
Factor 2: Care situation	8.04	55.37	6
Factor 3: Support situation	7.01	62.38	5

Varimax rotation was used to determine which variables belong to which factor and also to make the factors interpretable. Table 3 shows the three factors extracted from factor analysis using matrix rotation and the factor loading of each item.

**Table 3:** Items and factor loading related to the extracted factors

Items	Extracted factors		
	**Professional situation**	**Care situation**	**Support situation**
12. I protect the legal and moral rights of patients	0.64		
13. If the treatment is against professional values (such as justice, confidentiality, etc.), I will avoid participation	0.69		
14. I participate in research related to nursing	0.63		
15. I respect the privacy and confidentiality of the patient's information	0.75		
16. I cooperate with nursing organizations (Ministry of Health) to ensure the best standards of care in my practice	0.59		
17. I report any abuse or unethical behavior of colleagues to the appropriate regulatory authority	0.58		
18. I use available resources fairly in my professional performance	0.76		
19. I do my daily work activity by recognizing and introducing ethical issues and dilemmas in the profession	0.63		
1. I respect patients and their autonomy (such as the principles of freedom of choice)		0.59	
2. I do my work based on valid and up-to-date (new) scientific knowledge		0.78	
3. I protect the health and safety of the community		0.73	
4. I perform care, except in special cases, in accordance with professional standards		0.72	
5. I provide care individually and personally (for each patient), based on the principle of equality and without discrimination and prejudice		0.70	
8. I respect professional confidentiality		0.79	
6. I compensate for possible weaknesses and inefficiencies in the workplace			0.66
7. I should use ethical counseling in ethical dilemmas related to care matters			0.81
9. I review clinical documentation for quality (correctness and completeness)			0.60
10. I measure and evaluate a specific situation or problem, to benefit from the support of other colleagues			0.60
11. I apply the research results in my professional practice			0.72

**- Confirmatory factor analysis**

**Fig. 2:**
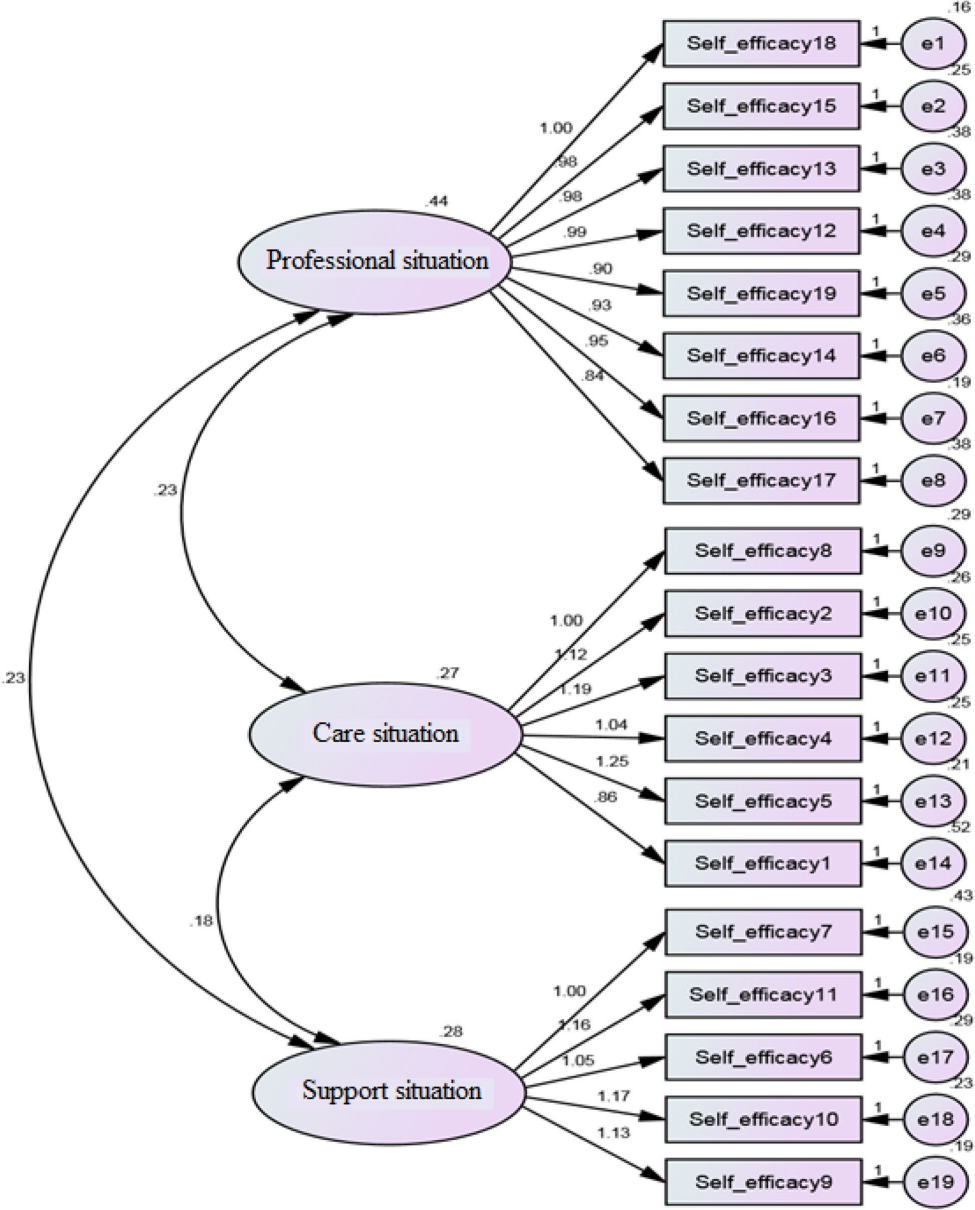
Results of confirmatory factor analysis in standard mode

The values of fit indices in the confirmatory factor analysis indicated the acceptable fit of the proposed model (Table 4, Fig. 2).

**Table 4:** Goodness of fit indices

**Indices**	*χ*^*2*^ (df)	RMSEA	CFI	NFI
Observed value	225.59 (149)	0.05	0.96	0.95

#### Reliability

Cronbach's α coefficient was used to determine the reliability of consistency. Cronbach's α coefficient was calculated for the entire questionnaire as 0.86, which indicates the acceptable reliability of the tool (Table 5). The intra-class correlation coefficient (ICC) value was 0.83 for the "Persian version of nursing profession self-efficacy", 0.87 for the professional situation subscale, 0.76 for the care situation subscale, and 0.72 for the support situation subscale. Hence, it indicates the acceptability and appropriateness of the reliability of temporal consistency or repeatability of the questionnaire (Table 5).

**Table 5:** Cronbach's α coefficient for the entire questionnaire and each dimension after determining validity

Dimensions	Frequency/number of items	Cronbach’s α	ICC
Professional situation	8 items (12, 13, 14, 15, 16, 17, 18, 19)	0.91	0.87
Care situation	6 items (1, 2, 3, 4, 5, 8)	0.86	0.76
Support situation	5 items (6, 7, 9, 10, 11)	0.87	0.72
The overall scale	19 items	0.86	0.83

## Discussion

It is important to use valid and reliable tools in any research. Invalid and unreliable tools may lead to false or questionable findings [38]. This study was conducted with the aim of translating and psychometrically evaluating the Persian version of "Nursing Profession Self-Efficacy Scale".

The translation is a process through which a message in the source language can be transferred to the target language. The basic issue in the use of foreign questionnaires is the correct translation and implementation of the culture of the research community. Foreign questionnaires provide the possibility of comparing the results with other countries in accordance with the culture of the target population [39]. The findings of the translation stage in this research indicated the acceptability of the translation of the original scale into Persian. In the study by Oh et al., which examined the validity and reliability of the same scale in Korea, the translated items were consistent in terms of meaning and the translation of the instrument was acceptable [20]. Therefore, good and culturally adapted translation of the NPSES creates an opportunity to compare concepts in the two target and reference societies.

The translated instrument should be revalidated with the target population due to the potential distortion of items during the translation process [39]. In the psychometric evaluation phase in determining the face validity, the Persian version of the Nursing Profession Self-efficacy Scale was evaluated as clear, suitable and satisfactory in terms of concept. Face validity is the degree of appropriateness of the appearance of the scale to collect the considered data from the point of view of the respondents [40]. In line with the present study, in Oh et al.'s study, at the face validity investigation stage, to identify items with unclear meanings and ambivalent understanding, the questionnaire was given to 28 nurses; after changing some phrases and terms by experts, the questionnaire was given to them again to finalize the scale, which was evaluated as appropriate and clear [20]. Taking the views of target users on the acceptability and validity of items into account is important, this can be achieved only through qualitative work with users [41].

The results of content validity investigation indicated the confirmation of content validity of the scale, and based on the calculated value of the content validity index, none of the items were deleted. In the present study, content validity was evaluated by 15 experts, but in Oh et al.'s study, content validity was evaluated by six experts in two stages. In the Korean version of this scale, none of the items were removed [20]. In Caruso's study, the average content validity index was 0.87 [18]. This evidence indicated the ability of the selected items to reflect the characteristics of the construct to be measured [42].

The output of the exploratory factor analysis suggested that according to the Eigenvalues, in total, 3 factors could be extracted and these three factors explained 62.38% of the total variance, which shows the desirability of the measurement. The values of fit indices in the confirmatory factor analysis also indicated the acceptable fit of the proposed model with the data. However, the original scale had two dimensions. The dimension of attributes of caring situations included 12 items and professionalism situations dimension included 7 items [18]. Consistent with the results of the present study, in Oh et al.'s study, after conducting exploratory and confirmatory factor analysis, this scale was finalized with three factors of professional, care, and support situation. In Oh et al.'s study, similar to the current study, the factor loading of all items was above 0.4 and none of the items were omitted [20].

Based on these findings, despite different cultures, it seems that there are similar attitudes towards professional and care situations in nursing environments in these countries. In both the original [18] and Korean [20] scales, in line with the results of the present study, items related to respect for patient autonomy, protection of community safety and health, care in accordance with professional standards, and care based on the principle of equality have been loaded in the care situation factor. Also, in both the original [18] and Korean [20] scales, avoiding participation in treatment contrary to professional values, participation in research, cooperation with nursing organizations, and reporting the unethical behavior of colleagues are loaded in the professional situation factor. In this study, consistent the original scale, compliance with professional confidentiality is included in the care situation dimension. Similar to the Korean scale, the item related to compensating for weaknesses, recognizing ethical dilemmas, and using ethical counseling for ethical dilemmas are loaded in the dimension of caring, professional and supportive situations, respectively. The rest of the items have been moved. It should be noted that differences in social norms and acceptance across cultures and generations can be the reason for moving items [20]. In other words, the difference in the characteristics of the participants and different cultural backgrounds are attributed to different nursing environments and may explain the differences in the structure of the factors [43].

Cronbach's α coefficient was 0.86 for the Persian version of the scale and the intra-class correlation coefficient was 0.83, which indicates the reliability of the instrument used. In Oh et al.'s study, Cronbach's α for the entire scale was 0.9 and intra-class correlation coefficient was 0.93 [20]. Finally, in Caruso's study, the Cronbach's α coefficient for the entire scale was 0.83 [18]. Reliability is a necessary condition for validity. According to the results, it can be argued that the Persian version of the scale has good internal consistency and stability.

### Limitations of the study

As one of the limitations of the present study, the convergent and discriminant validity were not investigated. In this study, only nurses working in public hospitals from one city were studied using convenience sampling, therefore, caution should be taken in generalizing the results. In addition, Nursing Profession Self-Efficacy Scale is a self-report measure that may be associated with social desirability bias. It can be pointed out that due to the newness of the tool and the development and validation of the original tool in English, the researcher faced the problem of lack of resources to obtain studies in this field for better discussion. Besides, since this tool has been psychometrically evaluated for the self-efficacy of nurses in Iran, care ought to be taken in using it for other languages and cultures.

### Implications for nursing and health policy

The Persian version of "Nursing Profession Self-Efficacy Scale" is acceptable for those working in the nursing profession according to the psychometric results. It appears that it has the necessary sufficiency to evaluate the self-efficacy of Iranian nurses [20]. It is important to know the level of self-efficacy of nurses considering its role in nursing practice, adherence to competency standards in clinical practice, and professional identity of nurses [43]. Consequently, by using this instrument, it is possible to have a more accurate evaluation of the self-efficacy of Iranian nurses. This scale can help to improve the awareness of nursing managers about Iranian nurses' self-efficacy and plan accordingly, to increase the self-efficacy of nurses. By taking the necessary measures to improve the level of nurses’ self-efficacy, we can help to implement the mission of nursing to improve patient care and increase the health level of the Iranian community.

## Conclusion

The results of this study showed that the Persian version of "Nursing Profession Self-Efficacy Scale" is fluent and understandable for nurses due to the absence of difficult words. The results of face validity and content validity of the Persian version of "Nursing Profession Self-Efficacy Scale" indicated that the instrument has a good appearance and is suitable for assessing nursing profession self-efficacy. Construct validity indicated the existence of three factors: professional situation, care situation, and support situation. Further, the reliability of the Persian version of the scale showed that this questionnaire has good internal consistency.

It is suggested to conduct more studies on the validity of this scale in other social contexts. Other studies should be conducted to reinforce the validity (the convergent and discriminant validity) of the Persian version of "Nursing Profession Self-Efficacy Scale", as well as to explore its association with health outcomes.

## Data Availability

The datasets used and/or analyzed during the current study are available from the corresponding author on reasonable request.

## Abbreviations

NPSES: Nursing Profession Self-Efficacy Scale
SCVI/Ave: Scale-level Content Validity Index/Average
EFA: Exploratory Factor Analysis
CFA: Confirmatory Factor Analysis
KMO: Kaiser–Meyer–Olkin
df: Degree of freedom
RMSEA: Root Mean Square Error of Approximation
CFI: Comparative Fit Index
NFI: Normed Fit Index
SPSS: Statistical Package for the Social Sciences
Amos: Analysis of Moment Structures
SD: Standard Deviation
*χ*^*2*^: chi-squared
ICC: Intra-class Correlation Coefficient
